# Examination of EMS Decision Making in Determining Suitability of Patient Diversion to Urgent Care Centers

**DOI:** 10.3390/healthcare7010024

**Published:** 2019-02-02

**Authors:** Gerard Carroll, Katelyn Levy, Richard Pescatore, Rick Hong

**Affiliations:** 1 Cooper Medical School of Rowan University, 401 South Broadway, Camden, NJ 08103, USA; carroll-gerard@cooperhealth.edu (G.C.); levyk8@rowan.edu (K.L.); hong-rick@cooperhealth.edu (R.H.); 2 Crozer-Keystone Health System, 1 Medical Center Blvd, Upland, PA 19013, USA

**Keywords:** emergency medical services, urgent care, diversion, crowding

## Abstract

Widespread use of Emergency Medicine Services (EMS) for non-emergency care has increased recently, causing overcrowding of the Emergency Department (ED). The increased availability of urgent care centers (UCCs), with their ability to see large numbers of unscheduled patients with more acute presentations, may offer a viable option for many EMS systems to divert non-emergent cases. Using a survey-based study combined with retrospective chart review, EMS provider ability to determine patient suitability for diversion to UCCs was assessed. Results indicated a rate of inappropriate diversion of 11.6%. UCCs may be an alternative option for EMS transport, however strict protocols with medical direction are needed.

## 1. Introduction

Widespread use of Emergency Medicine Services (EMS) for non-emergency care has increased recently, causing overcrowding of the Emergency Department (ED). A 2012 study showed that the proportion of EMS transports that were medically unnecessary has also increased from 13% to 17% [1]. A potential strategy to offload non-emergent patients from the ED would be to permit EMS personnel determination of patient suitability for transport to an alternative destination. Patient diversion to non-ED settings may also be a useful strategy for decreasing ED volumes in the setting of disasters. International and domestic studies have examined the possibility of patient diversion by EMS to alternate care settings [2,3,4]. However, a meta-analysis has provided inconclusive evidence on whether EMS providers can accurately determine non-emergent from emergent patients [4,5]. No recent study has addressed the question of appropriate diversion by EMS. Appropriate diversion may represent an effective means of limiting or eliminating excess cost and resource utilization.

Patient determination to seek care in an ED or another setting is multifactorial and includes limited access to primary care, patient-perceived urgency, convenience and a belief that their condition requires resources offered by a particular healthcare provider [6,7,8,9]. Urgent Care Centers (UCCs) offer expedited access to a healthcare provider and medical services to patients with non-life or limb threatening medical concerns [10]. Ninety-six percent of UCCs are open seven days a week and at least four hours a day. Ninety percent report a waiting time of less than thirty minutes to see a provider. Patient utilization of UCCs is also increasing with 89% of UCCs reporting an increase in patient volume in 2014 [11]. While it is not yet clear why patients choose to seek treatment in an ED versus a UCC, diversion of patients from an ED setting to a non-emergency medical setting such as a UCC may alleviate ED overcrowding [8,12].

This question has become timely with the rapid growth of UCCs, which may offer a new pathway for EMS diversion protocols [10]. In many places, even if EMS displayed the ability to divert patients safely, the ability to divert transport away from the ED has been restricted by the complexity and rigidity of clinic schedules, non-standardized capabilities of UCCs, and regulatory restrictions, including restricted insurer payments when patients are not transported to EDs. However, over the last decade the increased availability of UCCs, with their ability to see large numbers of unscheduled patients with more acute presentations, may offer a viable option for many EMS systems to divert non-emergent cases. 

The goal of this prospective survey-based study was to examine the ability of emergency medical technicians (EMTs) to decide whether patients could be appropriately and safely diverted from an ED to a UCC. As non-emergent activation of EMS continues to increase, appropriate diversion may represent an effective means of limiting or eliminating excess cost and resource utilization. Previous investigations have relied heavily on mock decision making and advanced life support evaluation, with a 2009 systematic review and meta-analysis finding profound heterogeneity among studies and calling for more research into the practice.

## 2. Materials and Methods 

We conducted a prospective survey-based study. EMTs transferring care of patients to the ED completed a brief survey regarding patient suitability for diversion to a UCC. Attending emergency physicians (EPs) also completed the same survey immediately following their initial history and physical examination of the patient. Concurrent medical record review was used to ascertain patient-level data, including age, gender, ED chief complaint and ED triage vital signs. Data were collected on patient encounters during local UCC operating hours. Both ED and EMS staff were blinded to the objective of the study. Our institutional review board approved this study. 

This study was performed from 5 July 2016 to 11 August 2016, during normal operating hours of local UCCs from Monday to Thursday, involving an inner-city Level 1 trauma center and tertiary care university hospital with an annual ED census of 75,000 visits. EMS crews were comprised of two EMTs trained in basic life support and state-certified as EMTs.

A convenience sample of adult patients greater than 18 years of age transported to the ED by an EMS agency was included in the study. All ED patients initially assessed by the Trauma Team or by Obstetrics in the Labor and Delivery unit were excluded due to the use of a different triage system employed for these patients in both the field and the hospital. 

The patient was initially assessed and managed by the treating EMT per protocol. Upon arrival to the ED, the survey was completed by the EMT after transfer of care to the ED. After initial ED evaluation and prior to the results of any diagnostic studies, the EP completed the same survey.

The answers from the EMTs and EPs regarding the survey question “Could this patient have been diverted to an urgent care center?” were tallied and compared for agreement. Through medical record review, demographic information including age and gender, chief complaint for ED visit, and ED triage vital signs were collected.

The primary outcome measure was the level of agreement between EMTs and EPs regarding diversion to a UCC. Secondary outcome measures were the level of under-triage by the EMTs as the percentage of cases in which the EMTs considered UCC disposition appropriate, but the EPs disagreed, and variables from chart review that might impact transport destination.

The level of agreement was demonstrated using both simple percent agreement calculation and Cohen’s kappa coefficient. Simple percent calculation was used to show the frequency of under-triage by the EMTs. Continuous data are presented as means with comparisons between groups performed using an independent *t*-test or Mann-Whitney U test. Values are presented with 95% confidence intervals. 

## 3. Results

Data were collected on 235 consecutive patient encounters, 233 of which were evaluated by both an EMT and an EP. Two patients eloped from the ED prior to EP evaluation. In Table 1, diversion to a UCC was deemed appropriate by EMTs in 45 cases (19.3%, 95% CI 14.6–24.8). Thirty-five instances (15.0%, 95% CI 10.9–20) of discordance between EMT and EP determination were recorded, with 27 encounters (11.6%, 95% CI 7.9–16.3) under-triaged by EMTs who deemed the patient appropriate for transport to a UCC but inappropriate by the EP. The simple percent agreement between EMTs and EPs was 85.0% with a Cohen’s kappa coefficient of 0.426 (95% CI 0.272-0.580), signifying moderate agreement (Table 2).

Demographic information, ED chief complaints, and ED triage vital signs for patients with disagreement between EMTs and EPs regarding UCC transport and for those with agreement for UCC transport are presented in Table 3, Table 4 and Table 5. Musculoskeletal concern (50%) was the most common chief complaint in cases in which both EMTs and EPs agreed with UCC transport. However, during disagreement, traumatic mechanisms (25%) were most commonly found when EMTs preferred ED transport, and gastrointestinal issues (42%) when EPs preferred ED transport. Table 6 compares data between patients with agreement for UCC transport and those under-triaged by EMTs for UCC transport from the perspectives of the EPs. Age is the only finding to be statistically significant, with a mean of 48.11 years for patients agreed upon by both EMTs and EPs for UCC transport and a mean of 58.07 for patients under-triaged by EMTs (*p* = 0.0477).

## 4. Discussion

A primary objective of any EMS system is to ensure delivery of patients to the proper resource using the quickest, most appropriate means of transportation possible. Traditional EMS systems are designed to provide ambulance transport to an emergency department for anyone who calls 911 [13]. As in the ED, triage is an important mechanism in the out-of-hospital arena, and its significance is increasing as EMS systems institute significant operational changes to improve availability and conserve resources [14]. An effective, safe triage process and the ability to access alternative definitive care sites offer meaningful cost containment strategies for EMS [15]. However, although the EMTs and EPs in this study did agree upon a large percentage of cases for appropriate evaluation at either the ED or a UCC, moderate agreement between EMTs and EPs is not acceptable to promote the practice of transporting patients to a UCC instead of an ED. Our key finding is the level of under-triage by EMTs, as described by those cases that the EMTs believed were appropriate for UCC evaluation but inappropriate by the EPs. In this study, even though EMTs and EPs agreed that 198 patients (85%) should be transported to either the ED or a UCC, there were 27 cases (11.6%) in which the EP disagreed with the EMTs decision to transport to a UCC. This is a relatively low, yet still unacceptably high, under-triage rate. This disagreement could lead to delay in care, with potential for a poor outcome by permitting EMS to transport to a lower level of care [4]. EPs also disagreed with eight cases (3.4%) in which EMTs would have opted to transport to the ED instead of a UCC. This type of over-triage may lead to an inefficient allocation of ED resources [16], but over-triage is preferred to prevent harm to the patient by transporting to a facility that provides a higher level of care [17]. In contrast to other studies, both EMTs and EPs agreed that 18 of the 233 (7.7%) cases may not need ED services, which may imply that the public is able to determine when ED services are appropriate.

Further review of the demographic information and ED triage vital signs showed a significant age difference between the patients with disagreement between EMTs and EPs and those with agreement for UCC transport. A lower mean age (*p* < 0.05) was noted in the patients with agreement for UCC transport compared to those for which EPs disagreed with EMTs who opted for UCC transport, which may imply that age is a risk factor for overall morbidity and mortality. Additionally, review of chief complaints suggested that musculoskeletal complaints were most common in the population for which both EMTs and EPs agreed to UCC evaluation. EMTs appeared to prefer ED evaluation for injuries resulting from a significant traumatic mechanism such as a motor vehicle accident and fall, and EPs preferred ED evaluation for gastrointestinal symptoms such as abdominal pain, nausea, vomiting, and diarrhea. These findings may imply that any protocols involving diversion from an ED should include age parameters, chief complaints, and traumatic mechanisms.

UCCs have become ubiquitous in many healthcare markets, yet lack uniformity in the levels of care they provide, and generally will only evaluate insured patients who do not need to pay steep up-front fees [15]. In the local region where this study was conducted, UCCs are staffed with a range of providers including advanced practice nurses (APNs), physician assistants (PAs), non-board eligible/certified physicians, internal medicine physicians, family medicine physicians, and EPs. The range of facility capabilities is broad as well, with some mimicking a traditional office practice, while others offer emergency procedures, lab tests, electrocardiograms, diagnostic radiology, and even intravenous therapy. With additional education to EMS on UCC capabilities and the implementation of protocols, UCCs may provide alternate destinations for EMS during times when EDs are significantly busy and overcrowded, particularly in the setting of disasters or public health emergencies such as a pandemic. UCCs may provide a more viable option than a primary care office visit or a non-emergent ED visit given their capability to manage more acute presentations without an appointment. However, a “multiple option decision point” (MODP) model of EMS to match patient need to appropriate resources would require rigorous medical direction [4,18], possibly even telephonic and on-site decision-making about the severity of a patient’s illness and disposition to support alternatives to hospital treatment [19]. Many studies have shown that EMS providers may not consistently determine whether a patient requires transport given the only option is to transport to the ED [20], however with the existence of UCCs, EMS providers may be more successful in diverting appropriate patients away from the ED.

Barriers to UCC diversion include patient insurance status, EMS billing practices for non-ED destinations, willingness of patients to be diverted, and the UCCs’ agreement to accept ambulance patients. Many EMS agencies are challenged by traditional economic models that lead to staff challenges and decreasing reimbursement in the setting of increasing call volume. Many agencies are exploring resident subscription models and opportunities to decrease out-of-service (OOS) times. In settings where availability of service in the local area outweighs service reimbursement, transport to the local UCCs might be a viable cost containment strategy to decrease total ED offloading time. Current challenges for EMS and our study results suggest numerous future research opportunities, including implementation of strict UCC transport criteria based on the local area’s UCC capabilities with the goal of reducing discordance between ED and EMS provider triage. 

## 5. Limitations

There are several limitations to this investigation. First, this was a convenience sample of patients who were transported to our ED during the study period. Our sample may not be fully representative of our ED population or other patient populations. This study was also only conducted at one center, and we excluded patients who were immediately cared for by the trauma and obstetrics services. Our exclusion of patients presenting to either service was intentional, as these patients are always considered emergent, requiring immediate evaluation by a specialty service and therefore unlikely to receive appropriate care at a UCC. Another limitation is that the surveys were not simultaneously completed by the EMTs and the EPs. Since patient condition may change from prehospital to ED assessment, the survey answers may differ due to the progression of care or illness. Additionally, while our site’s EPs are familiar with UCC capabilities as many work in UCCs as well, EMS providers may not be as knowledgeable. Finally, variation in UCC provider training (e.g., emergency medicine, family medicine) and different levels of care (e.g., physicians, nurse practitioners, physician assistants) suggests that decision to transport to a UCC will be dependent on individual capabilities and may differ based on local centers.

## 6. Conclusions

Although there was a high percentage of agreement between EMTs and EPs regarding appropriate transport to a UCC or ED without protocols or medical oversight in place, the number of cases that would have been under-triaged by EMTs for transport to a UCC creates concern that patients may not receive appropriate care on a routine basis. However, during a large-scale event or public health emergency in which ED resources are significantly limited, emergency planners may consider UCCs as an alternative option for EMS transport in the setting of strict protocols with medical direction.

## Figures and Tables

**Table 1:** Summary of survey diversion question data.

Transport Decision	Answer (Yes/No)	EP―Transport to UCC?		Total
		Yes	No	
EMT―Transport To UCC?	Yes	18	27	45
	No	8	180	188
	Total	26	207	233

EP: emergency physician; UCC: urgent care center; EMT: emergency medical technician.

**Table 2:** Agreement between EMTs and EPs.

Total Cases with Destination Agreement	198
Agreement with UCC Transport	18
Agreement with ED Transport	180
Total Cases with Destination Disagreement	35
Under-Triage	27
Over-Triage	8
Percent Agreement	85.0%
Cohen’s Kappa Coefficient	0.426
Inter-Rater Agreement	Moderate

**Table 3:** Presence of under-triage (EP disagreement with EMTs for UCC transport).

Subject #	Age	Gender	Chief Complaint	RR	HR	SBP	DBP
6	61	F	Altered Mental Status	16	105	130	70
13	52	M	Intoxication	18	67	126	85
18	68	F	Vomiting	18	79	183	90
21	64	M	Chest Pain	18	88	117	83
26	40	F	Hyperglycemia	22	104	128	75
36	68	M	Spasms	18	93	115	93
42	60	F	Swollen Finger	18	71	147	57
47	21	M	Vomiting/Abdominal Pain	14	80	164	72
62	68	M	Abnormal Labs	18	86	120	66
69	61	F	Abdominal Pain/Vomiting/Diarrhea	20	64	178	98
70	51	M	Tingling	16	78	137	90
73	55	F	Tongue Swelling	16	84	130	88
79	56	M	Back Pain	18	96	149	70
81	30	F	Abdominal Pain	18	91	141	96
83	70	M	Dizziness	18	113	101	69
111	66	F	Abdominal pain/Vomiting	16	73	176	109
113	90	F	Abdominal Pain/Upper Body Pain	16	74	132	68
121	48	F	Neck Pain/Swelling	20	75	140	79
133	54	M	Dizziness	16	94	159	85
134	88	F	Muscles Soreness	20	96	140	86
138	65	F	Shortness of Breath/Dizziness	24	121	145	82
152	60	M	Alcohol Intoxication	18	76	130	77
174	31	M	Motor Vehicle Accident	18	63	160	87
182	75	F	Pain	18	74	97	50
189	68	M	Nausea/Dizziness	16	88	132	82
216	70	F	Fatigue/Chest Pain	18	55	166	67
223	28	F	Abdominal Pain/Suicidal	16	63	104	73
Mean ($\bar{x}$)	58.07			17.85	83.37	138.78	79.52

**Table 4:** Presence of over-triage (EP disagreement with EMTs for ED transport).

Subject #	Age	Gender	Chief Complaint	RR	HR	SBP	DBP
27	44	F	Motor Vehicle Accident	18	92	126	78
61	23	F	Fall	16	84	94	55
65	67	F	Abnormal Labs	16	60	110	60
80	79	M	Hypoglycemia	18	76	129	64
105	19	F	Chest Pain	16	85	122	72
162	73	M	Shortness of Breath	20	112	123	81
200	65	M	Abdominal Pain	16	66	150	103
217	49	F	Mental Health Problem	18	110	149	104
Mean ($\bar{x}$)	52.38			17.25	85.63	125.38	77.13

**Table 5:** Agreement with UCC transport.

Subject #	Age	Gender	Chief Complaint	RR	HR	SBP	DBP
3	28	F	Asthma/Shortness of Breath	15	94	131	92
23	55	F	Leg Swelling	18	102	129	80
28	50	M	Ankle Pain	16	53	142	76
37	33	M	Alcohol Intoxication	16	110	121	83
43	40	M	Back Pain	22	91	153	106
53	90	M	Back Pain	14	68	140	88
55	55	M	Back Pain	16	68	130	74
64	28	F	Shortness of Breath	19	97	121	79
89	70	F	Bilateral Leg Pain	16	62	157	67
117	48	F	Chest Pain/Shortness of Breath	16	98	138	74
128	26	F	Shoulder Pain	16	78	144	122
136	29	M	Vomiting	18	85	160	108
139	60	F	Anxiety	26	99	176	110
144	24	F	Vomiting	16	98	115	73
160	60	M	Alcohol Intoxication	16	89	118	73
165	67	M	Shortness of Breath	18	66	133	84
211	40	F	Back Pain	18	99	129	89
215	63	F	Back Pain	18	98	162	102
Mean ($\bar{x}$)	48.11			17.44	86.39	138.83	87.78

**Table 6:** Mean ($\bar{x}$) differences in age and ED triage vital signs.

	Age	RR	HR	SBP	DBP
Agreement for UCC	48.11	17.44	86.39	138.83	87.78
Under-Triage	58.07	17.85	83.37	138.78	79.52
*p*-value	0.0477	0.2543	0.3421	0.9761	0.1416
